# Lumbosacral Osteomyelitis and Discitis with Phlegmon Following Laparoscopic Sacral Colpopexy

**DOI:** 10.7759/cureus.671

**Published:** 2016-07-05

**Authors:** Amanda V Jenson, M.D., Robert Scranton, Danielle D Antosh, Richard K Simpson

**Affiliations:** 1 Department of Neurosurgery, Houston Methodist Neurological Institute; 2 Division of Urogynecology, Houston Methodist Hospital

**Keywords:** osteomyelitis, discitis, sacral colpopexy

## Abstract

Lumbosacral osteomyelitis and discitis are usually a result of hematogenous spread; rarely it can result from direct inoculation during a surgical procedure. Bacteria may also track along implanted devices to a different location. This is a rare complication seen from pelvic organ prolapse surgery with sacral colpopexy. A 67-year-old female developed increasing lower back pain four months following a laparoscopic sacral colpopexy. Imaging revealed lumbar 5-sacral 1 (L5-S1) osteomyelitis and discitis with associated phlegmon confirmed by percutaneous biopsy and culture. The patient was treated conservatively with antibiotics, but required laparoscopic removal of the pelvic and vaginal mesh followed by twelve weeks of intravenous antibiotics. The patient has experienced clinical improvement of her back pain. This is an uncommon complication of sacral colpopexy, but physicians must be vigilant and manage aggressively to avoid more serious complications and permanent deficit.

## Introduction

Pelvic organ prolapse is a common disorder that affects women as they age resulting in loss of support of the vaginal walls. Sacral colpopexy is one of the most durable approaches to surgically manage vaginal vault prolapse where mesh is attached to the vaginal walls from an abdominal route and anchored to the anterior longitudinal ligament overlying the sacral promontory [1-2]. Sacral colpopexy is a more durable approach to manage prolapse and is known to be the gold standard treatment for advanced vaginal prolapse. Lumbosacral osteomyelitis and discitis are rare complications following sacral colpopexy. There are only three cases reported in the literature, from a spine surgeon’s perspective [3-5], which do not provide guidance on an appropriate course of management. We report a case of lumbosacral osteomyelitis and discitis with phlegmon in a patient following a sacral colpopexy procedure and provide a diagnostic and treatment algorithm.

## Case presentation

A 67-year-old woman with osteoarthritis on ibuprofen presented with the complaint of low back pain. Approximately four months prior she had undergone a laparoscopic-assisted vaginal hysterectomy with bilateral salpingo-oophorectomy, laparoscopic sacral colpopexy with polypropylene mesh, transobturator midurethral sling, and cystoscopy for stage IV complete uterovaginal prolapse. There were no intraoperative complications, and initially the patient did well after surgery. The patient reported that she had a history of osteopenia in the lumbar spine as well as lower back discomfort preoperatively. Lower back pain is a common presenting symptom in cases of pelvic organ prolapse. The lower back pain gradually increased after surgery with an acute exacerbation four months postoperatively leading to an evaluation by her urogynecologist and then neurosurgical consultation. The pain was exacerbated with movement and bending. The patient denied any fever, vaginal bleeding, radicular pain, or abdominal pain. She did have a small amount of clear vaginal discharge. Other surgical history included an appendectomy, laparoscopic cholecystectomy, and prior vaginal bladder suspension for prolapse in 2006. A detailed physical exam found a small area of exposed mesh fibers at the vaginal apex. There was no evidence of meningismus, spinal tenderness to palpation, or motor or sensory deficit. Laboratory studies for ESR and CRP were elevated at 65 and 0.62, respectively. The differential diagnosis included vertebral compression fracture, disc herniation, osteoarthritis, vaginal mesh infection, and discitis with or without vertebral osteomyelitis.

**Figure 1:**
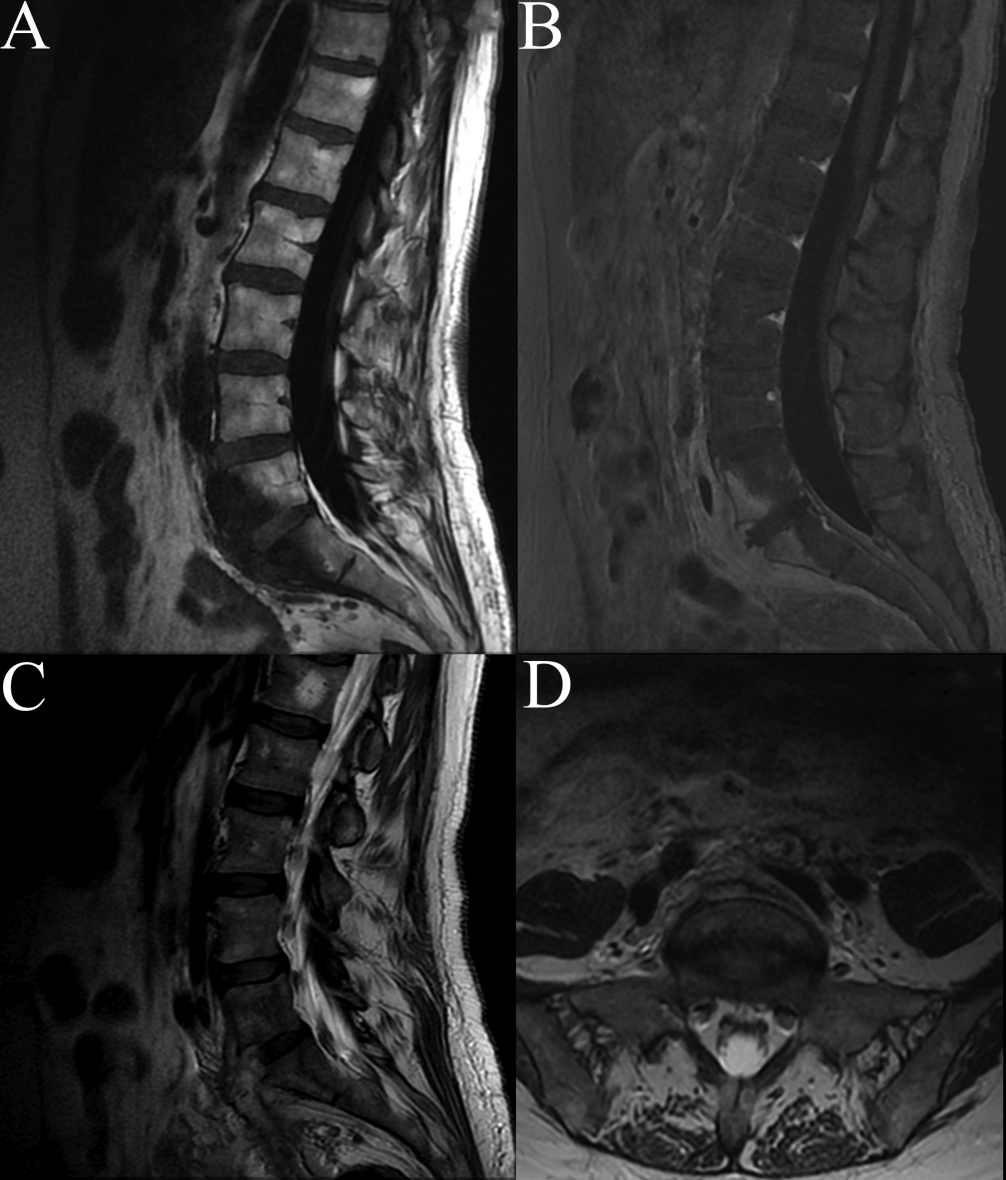
MRI Lumbar Spine Figure 1: MRI of lumbar spine with sagittal T1 (A) and contrasted T1 (B) with enhancement in the L5 and S1 bodies posterior to a hyperintense soft-tissue mass seen on T2 sagittal (C) and axial (D).

**Figure 2:**
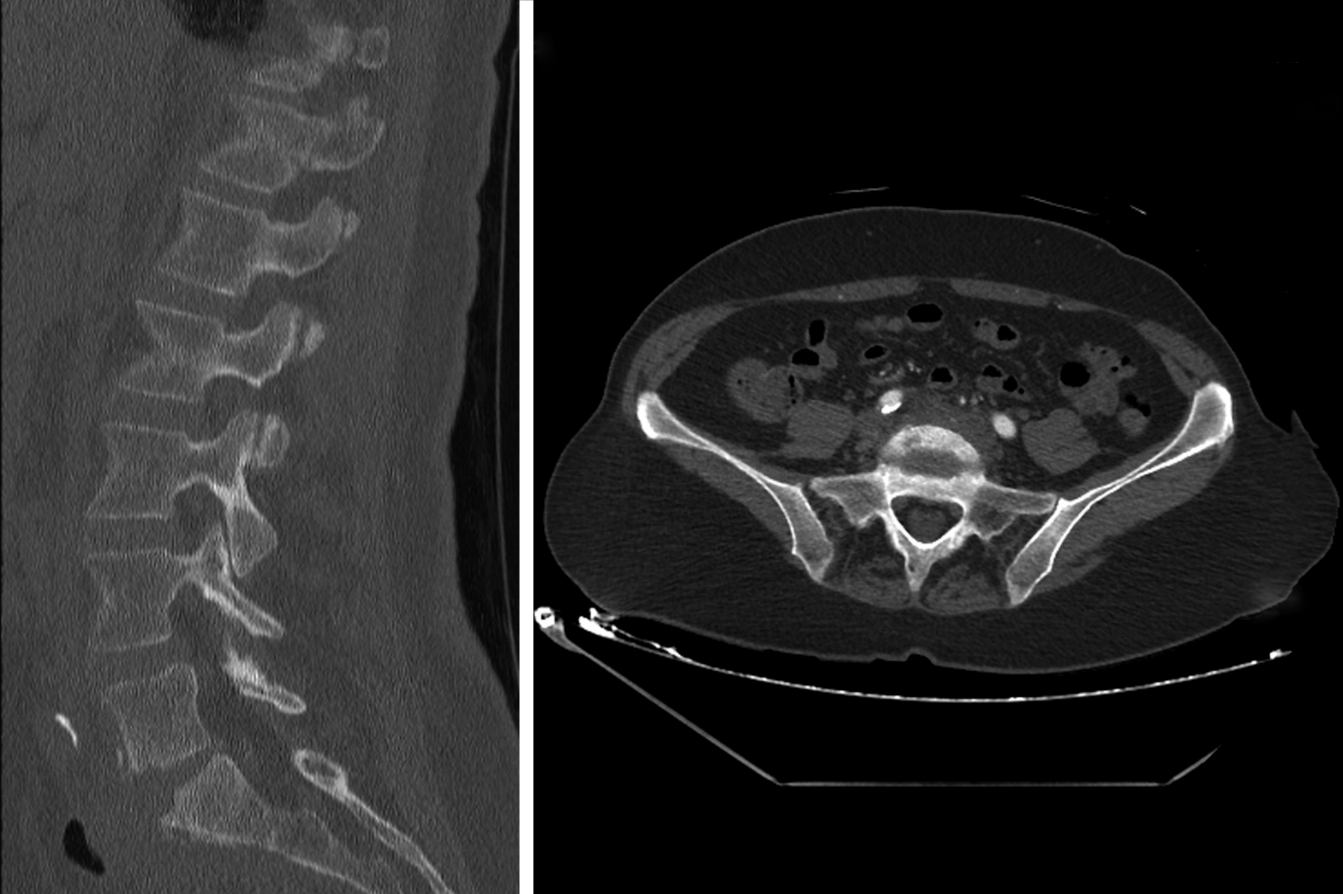
CT and CTA of Lumbar Spine Figure 2: Sagittal CT of lumbar spine with endplate destruction at L5 and S1 (left) and axial CTA pelvis (right) with iliac arteries embedded in soft tissue mass.

The patient was evaluated with a lumbar spine and pelvic MRI with and without contrast which showed T2 hyperintensity and T1 contrast enhancement in the body of L5 extending caudally through the L5-S1 disc space into the body of S1 (Figure 1). There was also an associated prevertebral soft tissue mass with T1 enhancement. A discrete fluid collection was not appreciated. A non-contrasted CT of the lumbar spine showed bony end-plate destruction suggestive of associated osteomyelitis (Figure 2). Imaging further focused the differential diagnosis making a vaginal mesh infection with secondary discitis and osteomyelitis most likely.

The patient was admitted for further workup and evaluation. An infectious disease specialist was consulted, and the neurosurgery service and gynecology service continued to follow the patient. A fluoroscopic-guided biopsy of the L5-S1 disk space and adjacent endplates was obtained and sent for culture before beginning empiric treatment with intravenous vancomycin and metronidazole. *Enterococcus faecalis, *vancomycin-resistant* Enterococcus gallinarum, *and* Bacteroides fragilis* were grown in culture. A peripherally inserted central catheter (PICC) was placed, and antibiotics were changed to Piperacillin/Tazobactam when culture susceptibilities became available. The patient remained afebrile without leukocytosis.  

On hospital day (HD) eight, further work up with a CT angiogram of the pelvis showed a phlegmon at L5-S1 involving the iliac vessels (Figure 2). Due to the severity of the osteomyelitis and due to concern for infected pelvic mesh with the bacterial culture results, on HD 13 the patient was taken to the operating room for removal of the infected pelvic and vaginal mesh by her urogynecologist. The plan was to remove all the mesh including the suture attachment to the sacral promontory without aggressive debridement of the sacral promontory due to the close proximity and risk of injury to the iliac vessels. The procedure included exploratory laparotomy with excision of the pelvic and vaginal mesh and cystoscopy with intraoperative placement of a right urethral stent. Findings included dense induration over the sacral promontory and induration of the peritoneum overlying the mesh in the right pelvic side wall without any purulent fluid collection. The mesh was still completely retroperitonealized, as performed in the index procedure. Extensive adhesions were dissected while mobilizing the sigmoid colon resulting in multiple serosal tears that were repaired by a colorectal surgeon. Surgical cultures of the sacral permanent sutures were positive for *E. Coli* and *Staphylococcus Aureus* sensitive to piperacillin/tazobactam. The patient was discharged home the following week on IV antibiotics scheduled for six weeks with follow-up through the infections disease service.

The patient was readmitted one week after discharge with acute onset of abdominal pain and brown vaginal discharge. CT of the abdomen and pelvis showed a fluid collection in the pelvis measuring approximately 7.5 x 5.6 x 4.2 cm suggestive of a hematoma or an abscess, confirmed as a hematoma on contrasted MRI. The ESR and CRP were remained elevated at 64 and 2.26, respectively, following a total of three weeks of antibiotics. The infectious disease service changed antibiotics to ertapenem and daptomycin secondary to elevated liver enzymes. She was discharged on HD two with her PICC line to continue IV antibiotics.  

**Figure 3:**
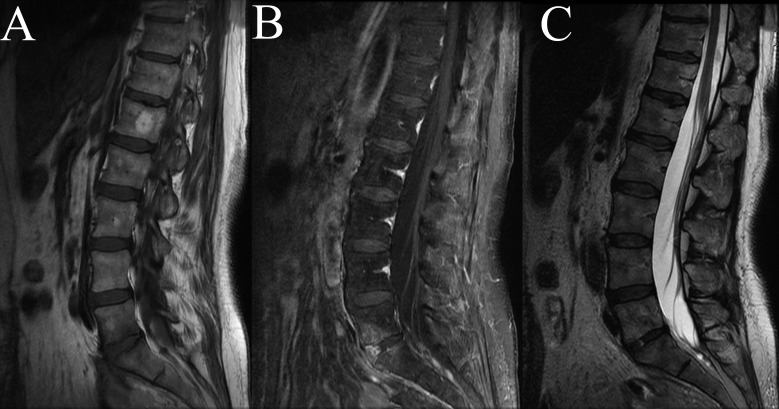
MRI of Lumbar Spine after Antibiotics Figure 3: Sagittal MRI of lumbar spine six weeks after completing a twelve-week antibiotic course with T1 (A), contrasted T1 (B) and T2 (C) sequences showing absence of previous T2 hyperintensity and enhancing scar anterior to L5-S1.

Approximately six weeks after IV antibitoics, MRI of the lumbar spine with and without contrast was repeated confirming previously seen discitis and osteomyelitis at L5-S1 but no further progression. ESR and CRP were decreased at 38 and 0.39, respectively. Antibiotics were continued for an additional six weeks, for a twelve-week total course. Four months after discharge, an MRI of the lumbar spine with and without contrast showed improving T2 hyperintensity and T1 contrast enhancement at the L5-S1 level (Figure 3). Clinically, the patient’s back pain resolved, and she has not experienced any new deficits or signs of infection. Informed consent was waived as no identifying features of patient were used. 

## Discussion

Vertebral osteomyelitis and discitis is a known but rare complication of sacral colpopexy, with few cases in the literature discussed from the perspective of a spine surgeon [3-5].

The most common etiology of vertebral osteomyelitis and discitis is the result of hematogenous seeding from a distant focus [6]. It can also occur due to direct inoculation from trauma, invasive spinal diagnostic procedures, surgery, or contiguous spread from adjacent soft tissue infection. Inadvertent placement of bone anchors into the L5-S1 disc space likely results in an L5-S1 inflammatory process with infection often tracking from an ulceration in the upper vaginal vault along the suspension sutures attached to the bone anchors [3]. Although this infection has been reported with the use of titanium bone anchors for mesh attachment over the sacral promontory, there have been case reports of this complication even when only permanent sutures are used, as in this patient case. This is evident on MRI when it clearly shows the nidus of spinal infection localized in the L5-S1 disc space and tracking along the mesh construct from the vaginal vault, as was apparent in our patient.

The 2015 Infectious Disease Society of America (IDSA) guidelines for treatment of vertebral osteomyelitis recommend an image-guided aspiration biopsy in all patients with suspected vertebral osteomyelitis, except when there is a bloodstream infection with S. aureus, S. Lugdunensis, or Brucella species [6]. In patients with a normal neurologic examination and stable hemodynamics, empiric antimicrobial therapy should be held until a microbiologic diagnosis is established [6]. However, we feel that if there is a significant concern for infection along the neural axis, and empiric therapy should begin immediately after obtaining a culture specimen and can be discontinued if an infection is later excluded.

Treatment includes antibiotic therapy based on the culture’s susceptibility for a minimum duration of six weeks [6]. Treatment of osteomyelitis may require surgical debridement of necrotic material and bone [7]. Surgical debridement is recommended when patients have progressive neurologic deficits, progressive deformity, spinal instability, or pain despite adequate antimicrobial therapy [6]. Despite the IDSA recommendations for progressive neurological deficit, our recommendation is that any level of neurological deficit should be evaluated by a neurosurgeon. Failure to decompress the spinal cord or nerve root with a recent deficit could potentially result in permanent disability. Surgical debridement with or without stabilization is also warranted if the patient has persistent or recurrent bloodstream infection (without alternative source) or worsening pain despite appropriate medical therapy [6]. If the patient is clinically improving with down trending inflammatory markers, then surgical debridement is of less benefit. A study was conducted on 21 patients with pyogenic vertebral osteomyelitis, all treated with titanium mesh cages and IV antibiotics postoperatively for a minimum of six weeks [8]. The indications for surgery included neurologic compromise, significant vertebral body destruction with loss of sagittal alignment, failure of medical treatments, and/or epidural abscess [8]. All patients had resolution of their infection with no instrumentation failures or signs of chronic infection [8]. In our practice experience, an ongoing infection is not a contraindication to spinal instrumentation. As for whether or not the pelvic mesh supporting the vagina should be removed, the urogynecologist and infectious disease specialist felt, with positive bacterial cultures of the disc, there was a high likelihood that the mesh was infected and needed to be removed. There have been case reports of treating discitis after sacral colpopexy successfully with IV antibiotics alone, although only in three of the eight reports published [2]. Furthermore, these patients were not followed long-term, and it is unclear if there was any recurrent infection or sequelae. 

Physicians must recognize the possibility of vertebral osteomyelitis and discitis in patients who have undergone sacral colpopexy. It is important to do a thorough past surgical history as the time from sacral colpopexy to the time of infection can have a wide range. Previous case reports show patients presenting from two weeks to four months [2, 5], but more remote cases as far as five to eight years after surgery have been reported [9]. Innovation has led to an increasing number of minimally invasive sacral colpopexy procedures using robotic-assisted laparoscopic techniques [7]. In a study done by Unger et.al., postoperative adverse events were compared between robotic-assisted laparoscopic sacrocolpopexy and conventional laparoscopic sacrocolpopexy in a cohort of 406 women [10]. Osteomyelitis was found postoperatively in 5.6% of the robotic-assisted laparoscopic sacrocolpopexy cases compared to 0% in the conventional laparoscopic sacrocolpopexy cases, P< .001 [10]. This is an interesting and worrisome finding and begs the question if this is merely a learning curve associated with the incorporation of new technology that will improve with experience. A prior case report published hypothesized that with robotic-assisted procedures, there is no haptic feedback leading to deeper placement of sutures through the ligament into the disc or bone [3]. This topic requires future consideration. 

## Conclusions

All physicians should have a high index of suspicion in patients who present with increasing low back pain after sacral colpopexy as this can be a warning sign of a potentially serious complication rather than a chronic complaint. This has become a significant issue spine surgeons should be aware of. The FDA has just reclassified surgical mesh used in the transvaginal repair of pelvic organ prolapse as a “Class III” device, generally reserved for high-risk devices, and now requires a premarket approval pathway. Our patient had a transabdominal insertion rather than transvaginal, and so the FDA reclassification does not apply in this instance. However, the result of mesh erosion is similar and must be considered in cases with transabdominal insertion. We recommend that patients be evaluated with imaging if there is new or worsening back pain or neurologic deficit after sacral colpopexy. If imaging shows any spinal or neurological involvement, surgical consultation would be indicated. Further, we recommend extreme caution when electing to operate on patients with a great degree of inflammation along the lumbar spine. Patients should receive a full six-week trial of aggressive antibiotic therapy prior to considering surgery unless there is a complicating factor such as neurologic deficit, exposed hardware, vascular compromise, or progression of the infection as suggested by an increasing white cell count, ESR, or CRP. Our patient proceeded to surgery before a full trial of antibiotics; however, she had infected mesh that had eroded through the vaginal vault, making an enduring cure with antibiotics unlikely without removal of the foreign body. The significant inflammation and scarring make surgery very risky with possible injury to the iliac vessels, ureters, and bowel. Although discitis and osteomyelitis is an uncommon complication after sacral colpopexy, it is important that physicians be aware of it and manage aggressively to prevent permanent injury.
