# Correction: Lumbosacral Osteomyelitis and Discitis with Phlegmon Following Laparoscopic Sacral Colpopexy

**DOI:** 10.7759/cureus.c5

**Published:** 2016-10-12

**Authors:** Amanda V Jenson, Robert Scranton, Danielle D Antosh, Richard K Simpson

**Affiliations:** 1 Department of Neurosurgery, Houston Methodist Neurological Institute; 2 Division of Urogynecology, Houston Methodist Hospital

**Title: **Lumbosacral Osteomyelitis and Discitis with Phlegmon Following Laparoscopic Sacral Colpopexy

**Authors:** Amanda V. Jenson, M.D., Robert Scranton, Danielle D. Antosh, Richard K. Simpson

**Original Citation: **Jenson, m.d. A V, Scranton R, Antosh D D, et al. (July 05, 2016) Lumbosacral Osteomyelitis and Discitis with Phlegmon Following Laparoscopic Sacral Colpopexy. Cureus 8(7): e671. doi:10.7759/cureus.671

The submitting and first author, Amanda V. Jenson, incorrectly included her medical degree initialism, M.D., in her name. This has been removed to produce the following corrected author list.

**Corrected Author List: **Amanda V. Jenson, M.D., Robert Scranton, Danielle D. Antosh, Richard K. Simpson

**Corrected Citation: **Jenson A V, Scranton R, Antosh D D, et al. (July 05, 2016) Lumbosacral Osteomyelitis and Discitis with Phlegmon Following Laparoscopic Sacral Colpopexy. Cureus 8(7): e671. doi:10.7759/cureus.671

